# Inhibition of signaling between human CXCR4 and zebrafish ligands by the small molecule IT1t impairs the formation of triple-negative breast cancer early metastases in a zebrafish xenograft model

**DOI:** 10.1242/dmm.023275

**Published:** 2016-02-01

**Authors:** Claudia Tulotta, Cristina Stefanescu, Elena Beletkaia, Jeroen Bussmann, Katsiaryna Tarbashevich, Thomas Schmidt, B. Ewa Snaar-Jagalska

**Affiliations:** 1 Institute of Biology, Animal Sciences and Health, Gorlaeus Laboratories, Leiden University, Einsteinweg 55, 2333 CC, Leiden, The Netherlands; 2 Physics of Life Processes, Kamerligh Onnes-Huygens Laboratory, Leiden University, Niels Bohrweg 2, Leiden 2333 CA, The Netherlands; 3 Leiden Institute of Chemistry, Gorlaeus Laboratories, Leiden University, Einsteinweg 55, 2333 CC, Leiden, The Netherlands; 4 Institute for Cell Biology, ZMBE, Von-Esmarch-Str 56, Muenster 48149, Germany

**Keywords:** CXCR4, CXCL12, IT1t, Triple-negative breast cancer, Metastases, Inter-species crosstalk, Xenograft, Zebrafish

## Abstract

Triple-negative breast cancer (TNBC) is a highly aggressive and recurrent type of breast carcinoma that is associated with poor patient prognosis. Because of the limited efficacy of current treatments, new therapeutic strategies need to be developed. The CXCR4-CXCL12 chemokine signaling axis guides cell migration in physiological and pathological processes, including breast cancer metastasis. Although targeted therapies to inhibit the CXCR4-CXCL12 axis are under clinical experimentation, still no effective therapeutic approaches have been established to block CXCR4 in TNBC. To unravel the role of the CXCR4-CXCL12 axis in the formation of TNBC early metastases, we used the zebrafish xenograft model. Importantly, we demonstrate that cross-communication between the zebrafish and human ligands and receptors takes place and human tumor cells expressing CXCR4 initiate early metastatic events by sensing zebrafish cognate ligands at the metastatic site. Taking advantage of the conserved intercommunication between human tumor cells and the zebrafish host, we blocked TNBC early metastatic events by chemical and genetic inhibition of CXCR4 signaling. We used IT1t, a potent CXCR4 antagonist, and show for the first time its promising anti-tumor effects. In conclusion, we confirm the validity of the zebrafish as a xenotransplantation model and propose a pharmacological approach to target CXCR4 in TNBC.

## Abstract (precis)

**Summary:** CXCR4-expressing human tumor cells respond to zebrafish cognate ligands and initiate metastatic events in a zebrafish xenograft model. The CXCR4 antagonist IT1t has promising tumor inhibitory effects.

## INTRODUCTION

CXCR4 is a chemokine receptor, first described in the early 1990s (Baggiolini et al., 1997; Herzog et al., 1993; Jazin et al., 1993; Nomura et al., 1993) and identified as a co-receptor for human immunodeficiency virus (HIV) entry (Feng et al., 1996). It is a seven-transmembrane G-protein-coupled receptor with a major role in physiological processes such as hematopoiesis (Nagasawa et al., 1996; Rosu-Myles et al., 2000), leukocyte trafficking (Day and Link, 2012; Sallusto and Baggiolini, 2008; Sallusto et al., 2000), cell migration and organogenesis during ontogeny (Bussmann and Raz, 2015), as well as pathological conditions like HIV pathogenesis (Vicenzi et al., 2013), WHIM syndrome (warts, hypogammaglobulinemia, infections and myelokathexis syndrome) (Gulino, 2003) and cancer (Balkwill, 2004; Chatterjee et al., 2014). Its cognate ligand is the homeostatic cytokine CXCL12 (Bleul et al., 1996; Oberlin et al., 1996) [also known as stromal cell-derived factor 1 (SDF-1)]. However, recent reports indicate that ubiquitin and macrophage migration inhibitory factor (MIF) can also bind to and signal through CXCR4 (Saini et al., 2010; Bernhagen et al., 2007; Saini et al., 2011; Pawig et al., 2015). Upon CXCL12 binding, CXCR4 triggers cell migration, proliferation and transcriptional control of downstream targets via G-protein-dependent or -independent mechanisms (Pawig et al., 2015). Dissociation of Gα and Gβγ subunits leads to Ca^2+^ release and activation of the PI3K/AKT and MAPK signaling pathways (Teicher and Fricker, 2010). CXCR4 dimerization occurs after ligand binding; subsequently, phosphorylation by JAK kinases takes place, followed by STAT signaling initiation in a G-protein-independent mechanism (Mellado et al., 2001; Vila-Coro et al., 1999). Moreover, initiation of β-arrestin signaling can take place, resulting in G-protein coupled receptor (GPCR) signaling blockade (Lefkowitz, 1998; Shukla et al., 2011) or ERK1/2 activation (Luttrell et al., 1999). CXCR4 activity is regulated by mechanisms of desensitization, through phosphorylation of the C-terminus and internalization, which is followed by degradation or recycling to the plasma membrane (Busillo and Benovic, 2007). Moreover, CXCL12 binds also to CXCR7 (Balabanian et al., 2005). However, differently from other chemokine receptors, CXCR7 does not signal through G proteins and acts as a ligand scavenger in a β-arrestin-mediated pathway (Rajagopal et al., 2010). Interestingly, a key role of the CXCL12-CXCR4-CXCR7 axis in collective tissue migration has been studied in zebrafish embryos. In the migration of the lateral line primordium, Cxcl12 scavenging by Cxcr7 leads to the formation of a self-generated gradient and cell migration after Cxcr4 activation, along tissues where Cxcl12 is homogeneously distributed (Boldajipour et al., 2008; Dona et al., 2013; Venkiteswaran et al., 2013).

A link between CXCR4 and cancer, in particular metastatic breast cancer, has been reported (Muller et al., 2001). CXCR4-expressing tumor cells preferentially colonize distant organs that secrete high levels of CXCL12, such as brain, lungs, lymph nodes, liver and bone marrow (Janowski, 2009). Among highly aggressive malignancies of the breast, triple-negative breast cancer (TNBC) is often associated with relapse and poor patient prognosis (Palma et al., 2015; Podo et al., 2010). Conventional hormone-based therapies are not applicable owing to the absence of expression of the estrogen and progesterone receptors, and Her2 gene amplification (Anders and Carey, 2009). Accordingly, surgery and chemotherapy are the main form of medical intervention and no targeted therapies are currently available (Wahba and El-Hadaad, 2015). Therefore, a better understanding of the biology of this aggressive breast carcinoma and the development of new therapies to reduce the high mortality rate are urgently needed. The bicyclam AMD3100, also known as plerixafor, is a CXCR4 antagonist and has been introduced in clinical trials to treat different tumor types, mainly leukemia and lymphomas (Ramsey and McAlpine, 2013). However, it has been reported to cause cardiotoxicity (Hendrix et al., 2004). AMD3100 also functions as an agonist for CXCR7 (Kalatskaya et al., 2009), which has been linked to breast cancer cell proliferation (Salazar et al., 2014). In addition, an anti-CXCR4 antibody is currently in clinical trials (Kuhne et al., 2013; Vela et al., 2015). More CXCR4 antagonists have been developed and *in vitro* as well as animal models are required to further explore clinical applications in patients.

Zebrafish is increasingly being used as an animal model for translational research in oncology (Amatruda et al., 2002; Barriuso et al., 2015; Ghotra et al., 2015). In particular, transparent zebrafish embryos allow following the behavior of fluorescent tumor cells in a living organism. Human cancer cells engrafted in the blood circulation of 2-day-old transgenic embryos, with fluorescently traceable endothelial (Lawson and Weinstein, 2002) and immune cells (Ellett et al., 2011; Renshaw et al., 2006), have been described to induce angiogenesis and form micrometastases in concert with immune cell interaction (He et al., 2012). Tumor angiogenesis and colonization of secondary tissues can be visualized in a short time period (2-6 days) in the small and fast-developing larvae. Although numerous discoveries have been made using zebrafish embryos as a xenotransplantation model, lack of knowledge about the communication between human and zebrafish cells has questioned its validity and partially limited its use.

Here, we report that the CXCR4-CXCL12 axis acts across zebrafish and humans and drives the formation of tumor micrometastases of human TNBC cells in zebrafish. Cell treatment with IT1t, a potent CXCR4 antagonist, and genetic silencing of *CXCR4* effectively inhibited early metastatic events *in vivo*. Therefore, using zebrafish as a xenotransplantation model, we propose a potential treatment to impair CXCR4 signaling and reduce the metastatic onset of TNBC.

## RESULTS

### TNBC cells display high *CXCR4* expression levels and increased metastatic behavior in a zebrafish xenotransplantation model

**Fig. 1.**
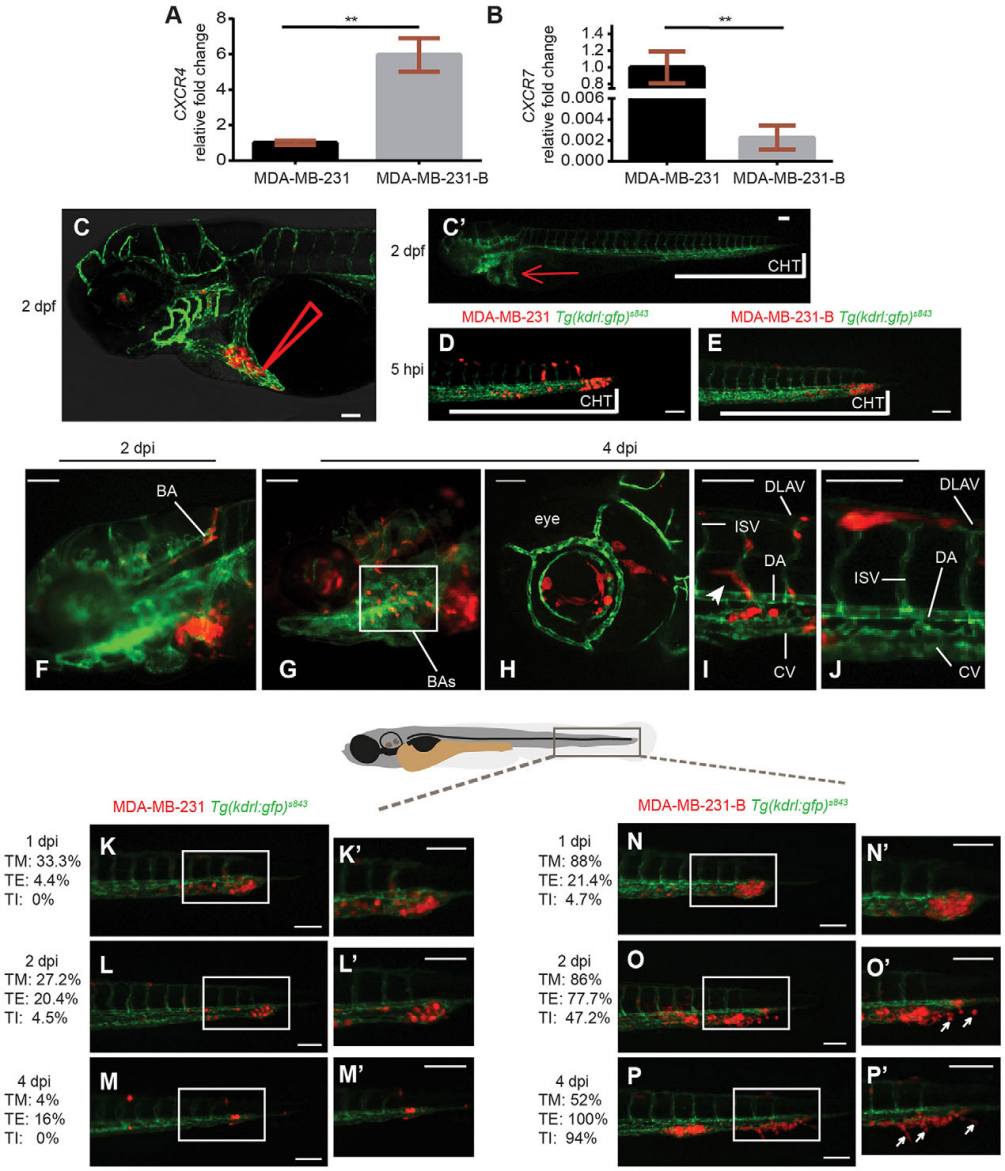
***CXCR4* expression levels correlate with metastatic potential in a zebrafish xenotransplantation model.** The bone clone (MDA-MB-231-B) expressed higher levels of *CXCR4* mRNA (A) and lower levels of *CXCR7* mRNA (B), compared to the parental cell line MDA-MB-231, originated from metastatic triple-negative breast cancer (TNBC) [unpaired *t*-test, (A) **P=0.0016, (B) **P=0.0019]. Upon engraftment into the duct of Cuvier (C,C′ red arrow) of 2-dpf zebrafish embryos (C′), MDA-MB-231 circulated in the vascular system (D), in a comparable manner to MDA-MB-231-B (E). Arrowhead in C represents the site of injection. CHT, caudal hematopoietic tissue. (F-J) Tumor cells disseminated throughout the embryo, in the head (F-H), the eye (H), the trunk and the tail (I,J), and extended filopodia at vessel branching points (I, arrowhead). BA, basilar artery; BAs, branchial arches; CV, caudal vein; DA, dorsal aorta; DLAV, dorsal longitudinal anastomotic vessel; ISV, intersegmental vessel. (K-P′) Over time, a weaker phenotype was detectable for the MDA-MB-231 cell line (K-M,K′-M′), whereas evident secondary tumor mass formation, extravasation and tail fin invasion persisted when MDA-MB-231-B cells were implanted (N-P,N′-P′). Arrows in O′ and P′ indicate invasive cancer cells that are not in contact with the endothelium and are found in the tail fin tissue, after extravasation. Images were acquired using a Leica TCS SPE confocal microscope with an HC PL FLUOTAR 10× DRY objective (0.30 N.A.) in panel C and with an HC APO 20× DRY objective (0.7 N.A.) in panel H. All other images were acquired using a Leica MZ16FA fluorescent microscope coupled to a DFC420C camera. Scale bars: 50 µm. Phenotype assessment was carried out at 1, 2 and 4 dpi, evaluating the ability of both cell lines to form a secondary tumor mass, to extravasate and to invade the surrounding tail fin. Images are representative of embryos injected with MDA-MB-231 and number of individuals was *n*=51 (5 hpi) (D), 45 (1 dpi) (K), 44 (2 dpi) (L) and 25 (4 dpi) (M) or with MDA-MB-231-B and number of individuals was *n*=44 (5 hpi) (E), 42 (1 dpi) (N), 36 (2 dpi) (O) and 34 (4 dpi) (P). Percentages relative to tumor mass (TM), tumor extravasation (TE) and tumor invasion (TI) are reported for each stage, for both MDA-MB-231 and MDA-MB-231-B cell lines.

We first characterized the expression profile of *CXCR4* and *CXCR7*, both chemokine receptors for CXCL12, in the TNBC line MDA-MB-231-B. Because this cell line derives from bone metastases of MDA-MB-231, after repeated engraftments into a murine host (Wetterwald et al., 2002), we used the parental line as a reference. We found that, compared to the original TNBC line, derived from human pleural metastases, the bone clone expressed higher *CXCR4* and lower *CXCR7* mRNA levels (Fig. 1A,B). Moreover, when compared *in vitro*, MDA-MB-231-B displayed a higher proliferation rate than the parental line (data not shown). To determine whether TNBC cells with increased *CXCR4* expression displayed a different behavior, we engrafted both MDA-MB-231 and MDA-MB-231-B in zebrafish. As previously reported (He et al., 2012), tumor cells were inoculated in the blood circulation of 2-days post-fertilization (dpf) embryos via the duct of Cuvier, a vein plexus that opens into the heart (Fig. 1C,C′). Fluorescent tumor cells derived from both cell lines entered the blood vessels and, at 5 hours post-injection (hpi), they were mainly found in the tail and trunk vessels of the *Tg(kdrl:EGFP)^s843^* zebrafish reporter line with green fluorescent vasculature (Fig. 1D,E). Injected embryos were examined by microscopy and embryos with 25-50 tumor cells hematogenously disseminating into the dorsal aorta (DA), caudal vein (CV) and vessel branches of the caudal hematopoietic tissue (CHT), in the region between the urogenital opening and the end of the tail, were selected for the experiment. Tumor cells spread through the embryo via blood circulation of the head, trunk and tail. Intravascular and perivascular cancer cells were found in the basilar artery (BA), branchial arches (BAs) and optic vessels in the head region (Fig. 1F-H), and in intersegmental vessels (ISVs), dorsal longitudinal anastomotic vessels (DLAVs) and the DA and CV in both the trunk and tail areas (Fig. 1I,J). Moreover, tumor cells were often positioned near vessel branching points (Fig. 1I), as to follow a path in a similar fashion to nascent lymphatic vessels, known to express *cxcr4a/b* receptors (Cha et al., 2012). Interestingly, *cxcl12a* and *cxcl12b* are expressed at these sites in developing zebrafish embryos (Cha et al., 2012; Fujita et al., 2011; Hess and Boehm, 2012). Highly aggressive cancer cells, adhering to the intravascular endothelium, initiated early metastatic events in the tail, sustaining tumor progression until 4-days post-implantation (dpi). In our model, in which tumor cells are inoculated directly into the blood circulation to study the formation of experimental micrometastases, bypassing initial modifications in a primary tumor mass, early metastatic events coincided with tumor foci formation and expansion, tumor extravasation, with adherence to the extravascular endothelium, and invasion. In line with previous work from our group, the tail fin region, in proximity of the CHT, a temporary site of hematopoiesis analogous to the fetal liver in mammalian development, was a preferential early metastatic site (He et al., 2012).

After reaching the vascular plexus that infiltrates the CHT, MDA-MB-231 and MDA-MB-231-B displayed divergent phenotypes. From 1 until 4 dpi, the parental line MDA-MB-231 showed a weakened behavior over time (Fig. 1K-M,K′-M′), whereas the bone clone induced increasingly aggressive phenotypes (Fig. 1N-P,N′-P′). The formation of a secondary tumor mass began at 1 dpi and was observed in 88% of the embryos engrafted with MDA-MB-231-B (*n*>40) (Fig. 1N,N′). In the MDA-MB-231 group, secondary tumors could be observed in 33.3% of the embryos (*n*>40) (Fig. 1K,K′). MDA-MB-231-B cells, with higher *CXCR4* expression, were found to progressively extravasate (from 21.4% at 1 dpi to 100% at 4 dpi), as well as to increasingly invade the surrounding tissue of the tail (from 4.7% at 1 dpi to 94% at 4 dpi) (Fig. 1N-P,N′-P′). Invasive cells were distinguished from extravasating cells once they were no longer in contact with the external wall of the endothelium and localized in the surrounding tail fin tissue (Fig. 1O′,P′). On the other hand, the MDA-MB-231 line, with lower *CXCR4* mRNA levels, displayed maximum extravasation at 2 dpi (20.4%), with a reduction at 4 dpi (16%). Invasive events were detected at 2 dpi (4.5%), whereas no invading cells were found in the tail fin at 4 dpi (Fig. 1M,M′). In conclusion, formation of a compact tumor structure, cancer cell extravasation, and invasion of the CHT and tail fin tissues increased over time in MDA-MB-231-B and decreased in MDA-MB-231. Taken together, our data show that the TNBC cell line MDA-MB-231-B displays high *CXCR4* expression and enhanced metastatic behavior in the zebrafish embryo.

### The CXCR4-CXCL12 signaling axis is cross-activated in zebrafish and humans

**Fig. 2.**
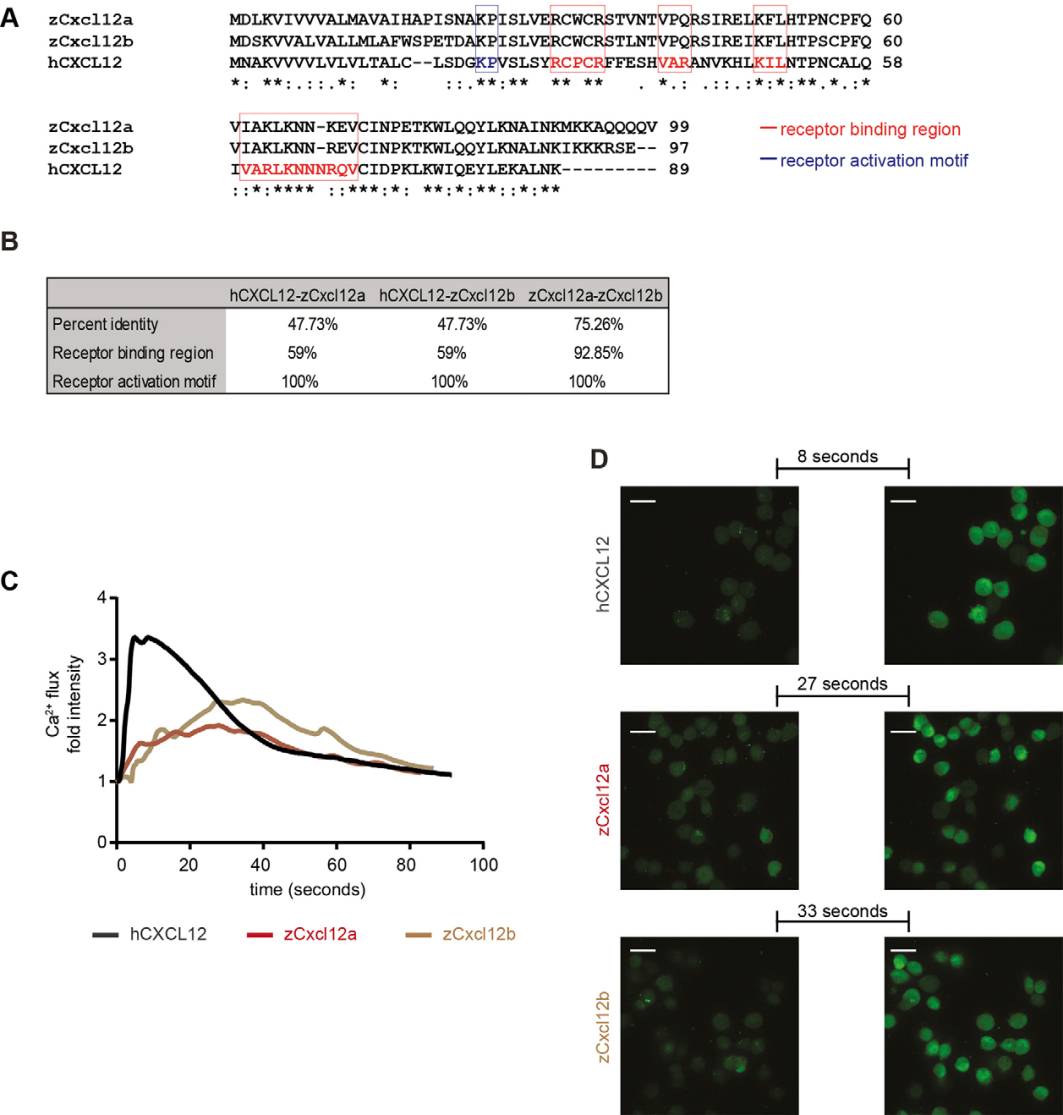
**Zebrafish Cxcl12 ligands activate CXCR4 signaling in human cancer cells.** Human CXCL12 was aligned to the zebrafish homologs using ClustalW (A). Amino acid residues were conserved in the receptor binding region and activation motif of CXCL12/Cxcl12 chemokines (A,B). Asterisks (*) represent fully conserved residues; colons (:) and periods (.) indicate positions at which residues share strong or weak similarity, respectively. (C,D) Human CXCL12-α and zebrafish ligands Cxcl12a and Cxcl12b induced calcium flux in MDA-MB-231-B cells, as detected by increased fluorescence intensity. The human ligand initiated an immediate response that extinguished rapidly (31 s to register half fluorescence intensity after the highest response), whereas the zebrafish ligands triggered a slower and prolonged signal induction (>55 s for zCxcl12a and >52 s for zCxcl12b to register half fluorescence intensity). (C) The fold intensity increase is calculated by normalization on fluorescence intensity correspondent to signal before response activation. (D) Frames show intensity before signaling activation was triggered by each ligand and at the highest peak of response. The time length to reach the strongest response is indicated.

Cancer cells expressing CXCR4 form distant metastases in secondary organs that produce high levels of CXCL12, in human specimens and murine models (Muller et al., 2001). Our initial findings showed that TNBC cells initiating early metastatic events in the zebrafish xenotransplantation model express high levels of CXCR4. To establish whether CXCR4 sustained tumor metastatic properties in a Cxcl12-dependent manner, we first assessed whether the CXCR4-CXCL12 axis acts across zebrafish and human. Two *cxcl12* genes, *cxcl12a* and *cxcl12b*, are found in zebrafish, following duplication events during teleost evolution. In a multiple alignment (Fig. 2A), human CXCL12 (α-isoform) shared 47.73% identical residues with both zebrafish Cxcl12a and Cxcl12b, whereas 75.26 was the percentage of identity between zebrafish Cxcl12 paralogs. Pair-wise sequence alignment showed 59% identity on residues involved in receptor binding, when the human ligand was compared to each zebrafish homolog, and 92.85% between Cxcl12a and Cxcl12b. Full identity in the motif involved in receptor activation was found (Fig. 2B). Therefore, considering the level of conservation, we verify that the CXCR4 signaling on human tumor cells is activated by both zebrafish Cxcl12 ligands. For this purpose, Ca^2+^ flux was measured. Whereas human CXCL12 (100 nM) failed to induce Ca^2+^ mobilization from intracellular storage into the cytoplasm in the parental line MDA-MB-231 (Fig. S1), time-lapse microscopy revealed that calcium sensor fluorescent signal intensity increased when MDA-MB-231-B cells were stimulated with either the human or the zebrafish ligands. In the bone clone, human CXCL12 elicited a response that increased and decreased rapidly. Zebrafish Cxcl12a and Cxcl12b triggered a slower but still significant response, in a non-synchronized fashion. In addition, the fluorescent signal gradually faded and failed to extinguish at once (Fig. 2C,D and Movies 1-3). Hence, we show that zebrafish Cxcl12 ligands trigger CXCR4 signal activation in human TNBC cells.

**Fig. 3.**
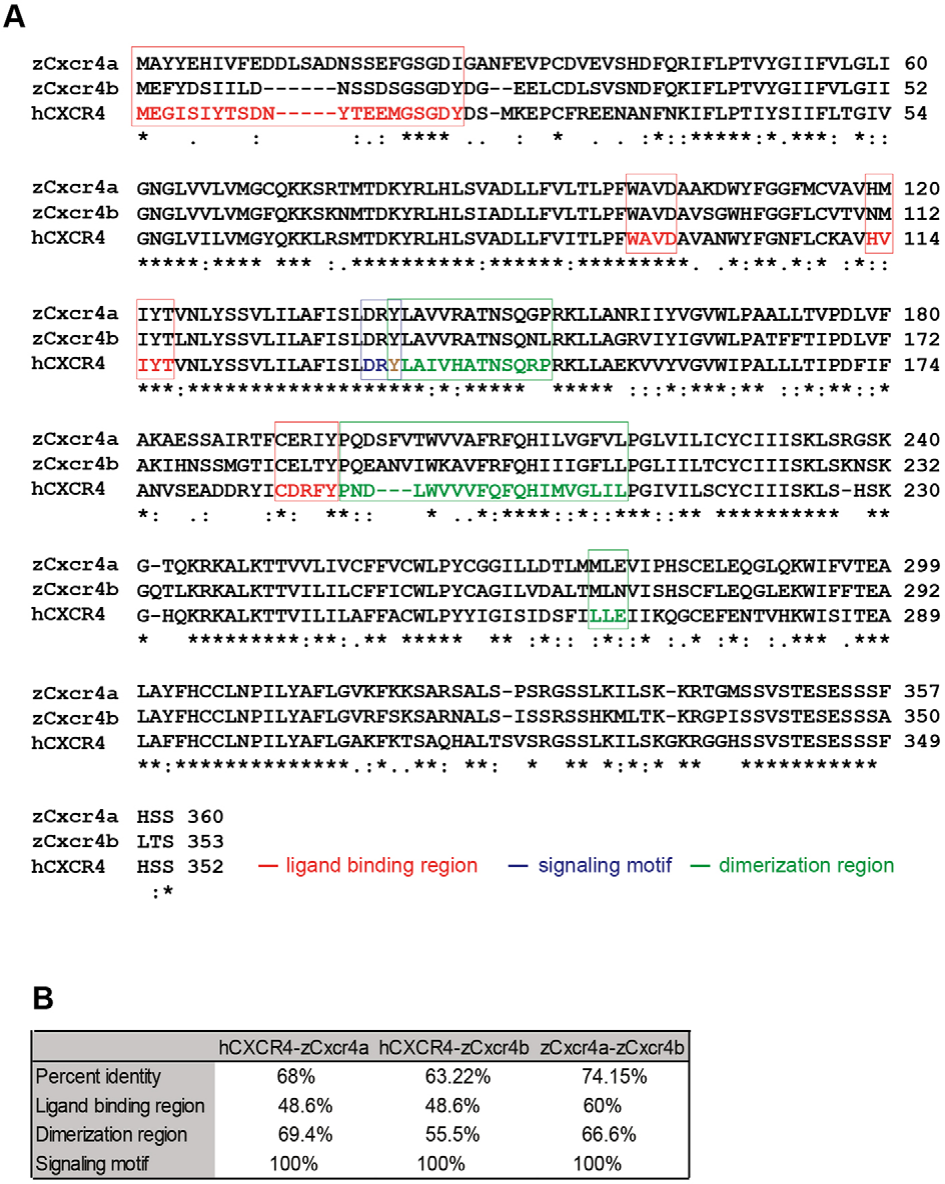
**CXCR4 alignment shows similarity between human and zebrafish proteins.** Human CXCR4 was aligned to the zebrafish homologs using ClustalW (A). Asterisks (*) represent fully conserved residues, colons (:) and periods (.) indicate positions at which residues share strong or weak similarity, respectively. The tyrosine (Y) in brown belongs to both the signaling motif and dimerization region. Amino acid residues were conserved in the ligand-binding region, and dimerization and signaling motifs in the CXCR4/Cxcr4 receptors (B).

**Fig. 4.**
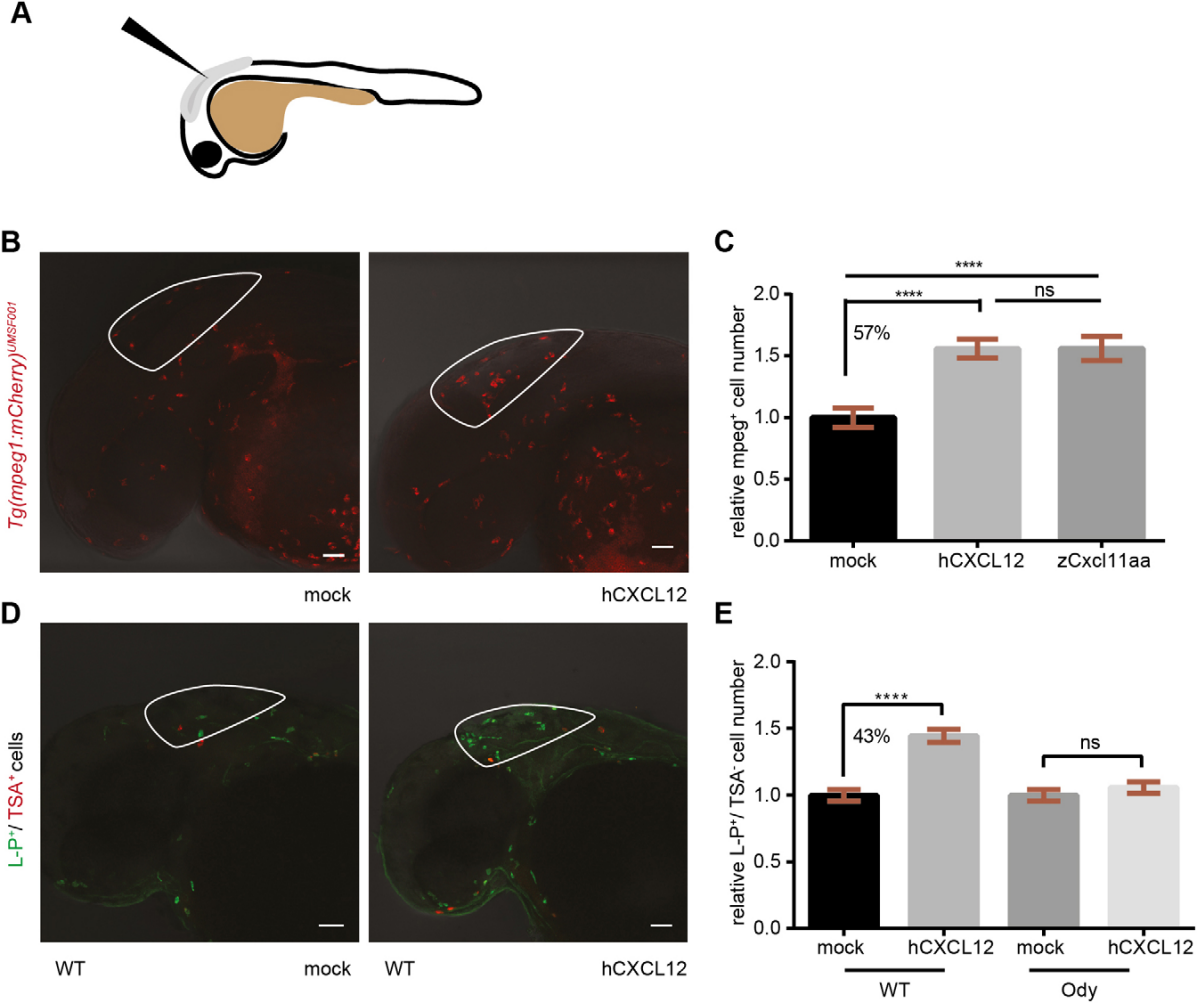
**Human CXCL12 triggers zebrafish macrophage migration in a Cxcr4-dependent manner.** (A) Scheme of a 30- to 32-hpf embryo and injection site are shown. (B,C) Zebrafish macrophages were found to be responsive to human CXCL12 (0.4 mg/ml) 3 h after injection into the hindbrain ventricle (HBV) of 30- to 32-hpf embryos. No increase in macrophage number occurred when a mock solution (water) was inoculated. Zebrafish Cxcl11aa (1.5 mg/ml) was used as a positive control (C). Data in C are pooled observations from two independent experiments (*n*=55 in mock; *n*=48 in hCXCL12; *n*=57 in zCxcl11aa). (D,E) Macrophages were recruited by human CXCL12 in a Cxcr4-dependent manner: a higher number (43%) of L-P^+^/TSA^−^ cells was found in the HBV compared to the mock-injected group in wild-type (wt) siblings (D,E), whereas no differences were detected in the *cxcr4b^−/−^* (*ody*) mutants (E). In B, mCherry-expressing macrophages are recruited by hCXCL12 as in D, where L-P staining combined to TSA detection is used to distinguish macrophages (L-P^+^/TSA^−^) from neutrophils (L-P^+^/TSA^+^). ****P<0.0001, ns *P*>0.05 one-way ANOVA, Bonferroni post-hoc test. Data in E are pooled observations from five independent experiments (*n*=171 in mock/wt; *n*=180 in hCXCL12/wt; *n*=139 in mock/*ody*; *n*=160 in hCXCL12/*ody*).

Next, we investigated whether human CXCL12 activates zebrafish Cxcr4. As for the Cxcl12 ligands, two Cxcr4 receptors have been described in zebrafish, Cxcr4a and Cxcr4b. ClustalW (Goujon et al., 2010; Larkin et al., 2007) alignments of the human CXCR4 with the zebrafish Cxcr4a and Cxcr4b (Fig. 3A) showed a percentage of identity equal to 68 and 63.22 on whole sequence, respectively (Fig. 3B). When ligand-binding regions were considered, the pair-wise identity reached 48.6% (CXCR4-Cxcr4a and CXCR4-Cxcr4b), whereas, in the dimerization regions, 69.4% (CXCR4-Cxcr4a) and 55.5% (CXCR4-Cxcr4b) of the residues were identical. Moreover, the signaling motif was 100% conserved (Fig. 3B). In addition, the zebrafish Cxcr4 paralogs displayed 74.15%, 60%, 66.6% and 100% identity at the whole-sequence level, ligand-binding and receptor-dimerization regions, and signaling motif, respectively (Fig. 3B). Besides partial redundancy, Cxcl12 and Cxcr4 zebrafish paralogs seemed to play distinct functions and to have a different spatial expression during embryo development, as reviewed (Bussmann and Raz, 2015). In particular, *cxcr4b* is found to be expressed by myeloid cells (Walters et al., 2010). To verify whether inter-species crosstalk exists between human ligands and zebrafish receptors, human recombinant CXCL12 (0.4 mg/ml) was injected in the hindbrain ventricle (HBV) of 30-32 hours post-fertilization (hpf) *Tg(mpeg1:mCherry)^UMSF001^* embryos, where macrophages are fluorescently labeled (Fig. 4A). A 57% increase in the number of cells that migrated to the site of injection was observed compared to the mock-injected group, and in a similar fashion to the zebrafish chemokine Cxcl11aa, previously shown to be a chemoattractant for this class of phagocytes (Torraca et al., 2015) (Fig. 4B,C). Furthermore, macrophage motility towards the human CXCL12 (α-isoform) was found to be Cxcr4-dependent. Macrophages did not respond to the human CXCL12 in the *ody* mutant line, with a non-functional Cxcr4b receptor. Injection of the human ligand in the HBV led to a 43% increase in macrophage number compared to the water-injected group, in the wild-type (wt) siblings, whereas no differences in mean cell number was detected when CXCL12- and mock-injected groups were compared in the *ody* mutants (Fig. 4D,E). Moreover, we excluded a possible Cxcr4b-dependent alteration of basal motility and total macrophage number in *ody* mutants compared to wild-type siblings (Fig. S2). In conclusion, zebrafish is a valuable *in vivo* model to study human cancers, particularly focusing on the interaction between cancer and host stromal cells, because human attractants trigger zebrafish cell migration and zebrafish ligands are sensed by human cells.

### Zebrafish Cxcl12-sensing by human CXCR4 receptor sustains TNBC cancer burden in zebrafish larvae

**Fig. 5.**
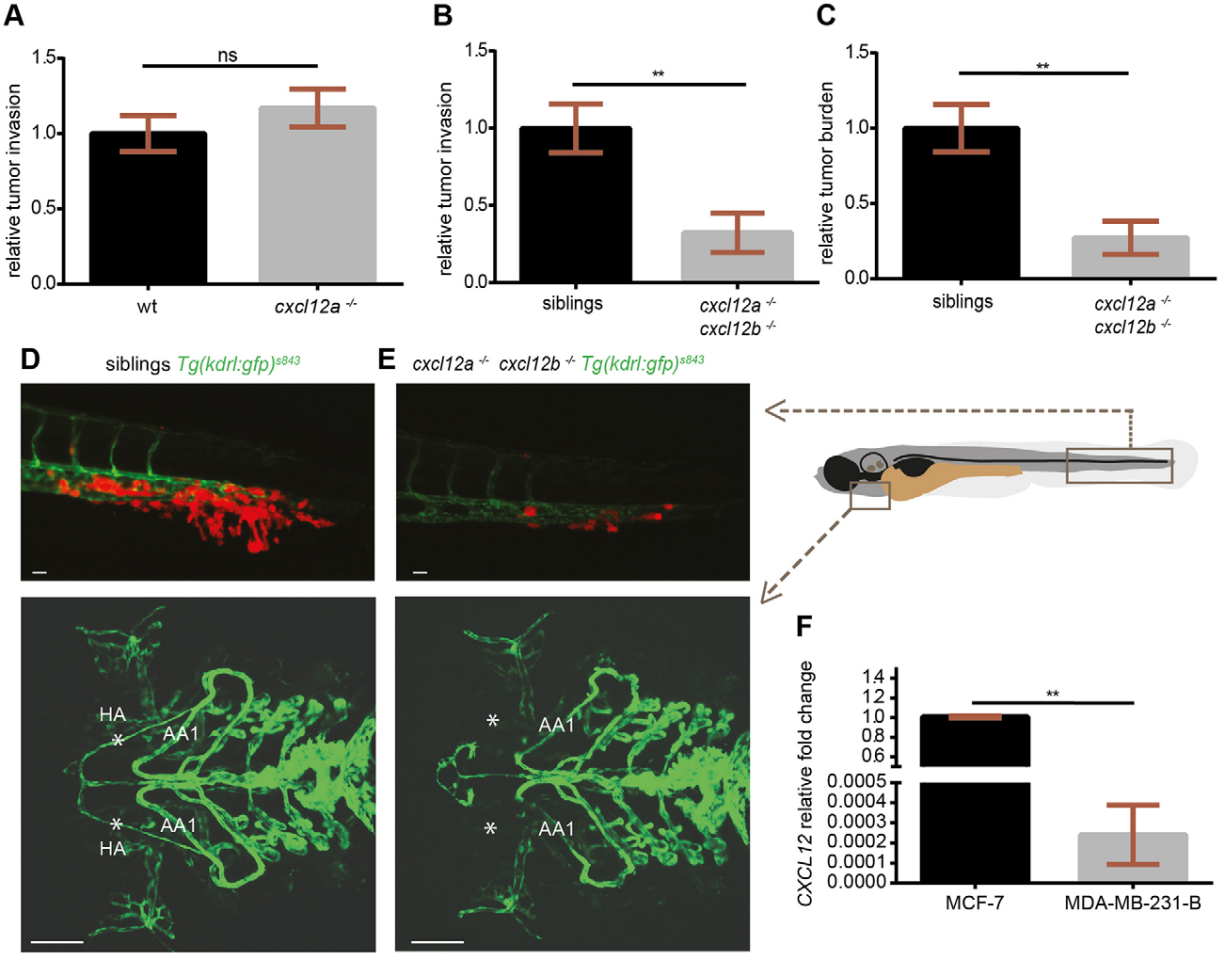
**CXCR4-expressing TNBC cells fail to initiate metastatic events in *cxcl12a*- and *cxcl12b*-null zebrafish mutants.** (A) No differences in tumor cell invasion were found in the *medusa* (*cxcl12a^−/−^*) mutants, compared to wild-type (wt) siblings. (B,C) Breast cancer cells failed to form micrometastases in 4-dpi zebrafish embryos deficient for both *cxcl12a* and *cxcl12b* ligands, whereas tumor invasion (B) and tumor burden (C) occurred in the *cxcl12a^−/−^/cxcl12b^+/+^* and *cxcl12a^−/−^/cxcl12b^+/−^* siblings. Unpaired *t*-test: (A) ns, *P*>0.05 (wt: *n*=73; *medusa*: *n*=66), (B) **P=0.0012 and (C) **P=0.0033 (*n*=11 in each group). Graphs in A-C are cumulative of two independent experiments. (D,E) Top panels: MDA-MB-231-B breast cancer cells were highly invasive in the siblings, whereas few tumor cells remained in the metastatic region in zebrafish that were mutant for both ligands. Images were acquired using a Leica MZ16FA fluorescent microscope coupled to a DFC420C camera. Bottom panels: vessel connections are compared to distinguish siblings and double mutants: the hypobranchial arteries (HA), indicated by asterisks, failed to connect to the mandibular arch (AA1) in the *cxcl12a^−/−^/cxcl12b^−/−^* larvae. Images were acquired using a Leica TCS SPE confocal microscope with an HC PL FLUOTAR 10× DRY objective (0.30 N.A.). Scale bars: 50 µm. (F) *CXCL12* expression is lower in the MDA-MB-231-B compared to MCF-7 breast cancer cell line. Unpaired *t*-test, with Welch's correction. **P=0.006. qPCR was performed on two biological replicates.

The tumor microenvironment plays a crucial role in the establishment of a favorable niche for the onset of cancer metastasis, and the CXCR4-CXCL12 axis, among other signaling pathways, guides the communication between tumor and microenvironment. We showed that crosstalk between human and zebrafish receptors and ligands (CXCR4/Cxcl12 and Cxcr4/CXCL12) occurs *in vitro* and in the zebrafish embryo model. Hence, we investigated whether CXCR4-expressing TNBC cells initiated early metastatic events *in vivo* after sensing zebrafish Cxcl12 ligands. MDA-MB-231-B cells were engrafted in the blood circulation of zebrafish embryos carrying null mutations in *cxcl12a* or *cxcl12b*. MDA-MB-231-B showed reduced localization in the head (BA and BAs) and trunk (ISVs) in larvae with a non-functional Cxcl12a (Fig. S3). However, tumor invasion developed similarly in *cxcl12a^−/−^/Tg(kdrl:EGFP)^s843^* larvae and wt siblings at 4 dpi (Fig. 5A). The same effect was observed in the *cxcl12b^−/−^/Tg(kdrl:EGFP)^s843^* mutants and wt siblings at 2 and 4 dpi (Fig. S4A). Like tumor invasion, also the overall micrometastasis burden in the tail fin was the same in wt, *cxcl12a^−/−^/Tg(kdrl:EGFP)^s843^* and *cxcl12b^−/−^/Tg(kdrl:EGFP)^s843^* at 2 dpi (Fig. S4B,C). However, the response of tumor cells in the Ca^2+^ assay to both zebrafish Cxcl12a and Cxcl12b supports the hypothesis that human tumor cells sense the Cxcl12a ligand in a *cxcl12b* mutant and the Cxcl12b ligand in a *cxcl12a* mutant. Therefore, in this scenario, tumor invasion and tumor burden could still occur in each single-ligand mutant line. Hence, the xenogeneic implantation was performed in the *cxcl12a^−/−^/cxc12b^−/−^/Tg(kdrl:EGFP)^s843^* double-mutant embryos. For this purpose, the *cxcl12a^−/−^/cxc12b^+/−^/Tg(kdrl:EGFP)^s843^* family was in-crossed and tumor engraftments were performed in the siblings of the F1 generation (experimental groups were blinded). Tumor burden and tumor invasion were significantly decreased in the double mutants compared to *cxcl12a^−/−^/cxc12b^+/−^/Tg(kdrl:EGFP)^s843^* and *cxcl12a^−/−^/cxc12b^+/+^/Tg(kdrl:EGFP)^s843^* siblings at 4 dpi (Fig. 5B,C and D,E, top panels). Mutant larvae for both ligands were distinguished from the siblings by screening for abnormal formation of the hypobranchial arteries at 6 dpf (Fig. 5D,E, bottom panels). Therefore, we suggest that the CXCR4-CXCL12 axis functions in a paracrine fashion across species and is responsible for driving the formation of TNBC micrometastases in zebrafish. Importantly, a potential role of the human CXCL12 autocrine loop in driving the formation of TNBC micrometastases *in vivo* is unlikely. *CXCL12* expression levels were undetectable in the parental line MDA-MB-231 (data not shown) and significantly lower in MDA-MB-231-B compared to the Luminal A (*ER*^+^, *PR*^+/−^, *Her2*^−^) MCF-7 breast cancer cell line (Fig. 5F). Accordingly, a very low expression of *CXCL12* in MDA-MB-231-B argued against the generation of a potential autocrine loop to activate the receptor CXCR4. Taken together, the onset of early metastatic events in this experimental system is enhanced by the CXCR4-CXCL12 axis in a zebrafish xenotransplantation model in which human tumor cells respond to zebrafish ligands.

### The CXCR4 antagonist IT1t reduces the formation of TNBC early metastases *in vivo*

**Fig. 6.**
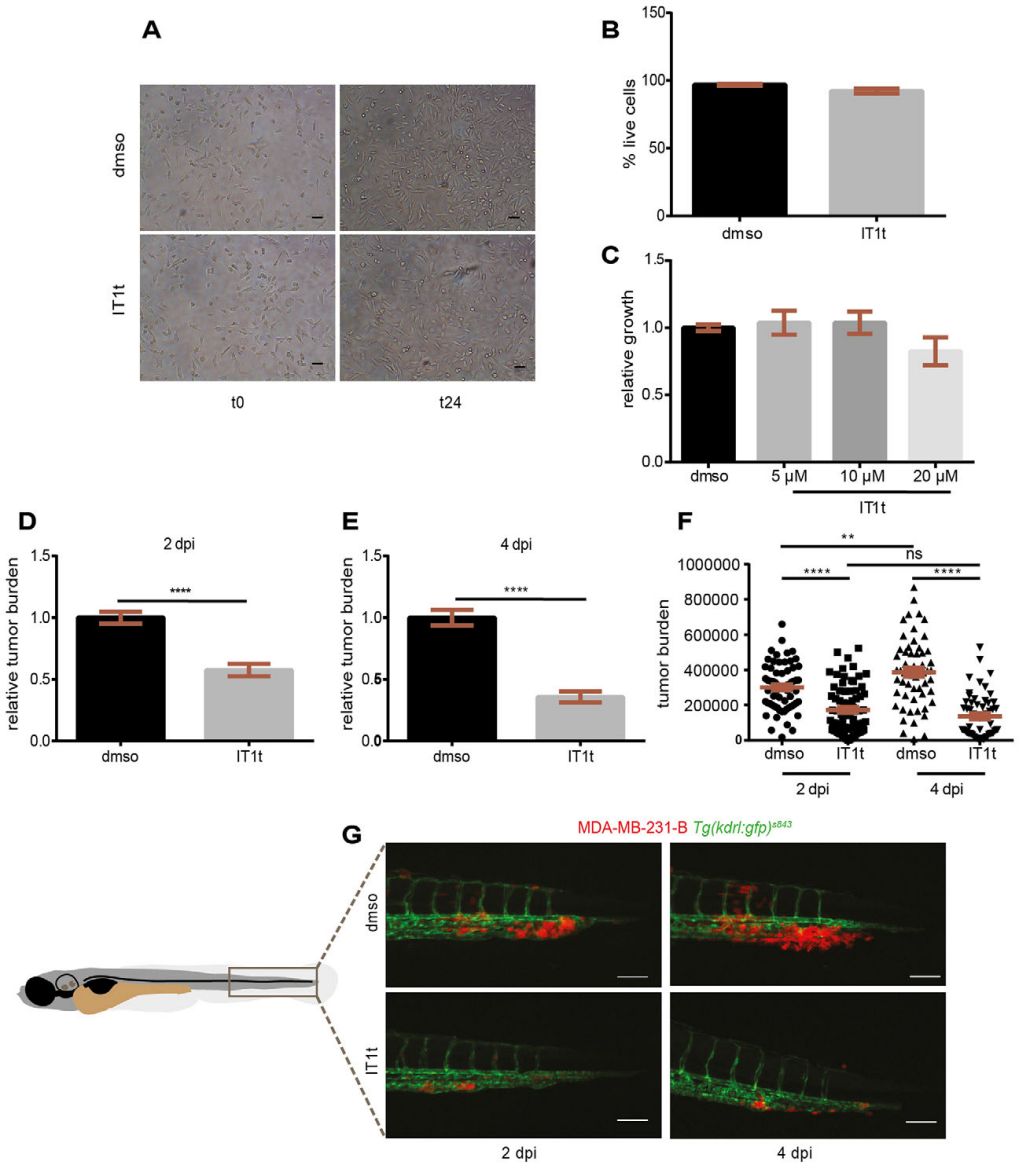
**The CXCR4 antagonist IT1t reduces metastatic tumor burden *in vivo*.** IT1t (20 µM) was applied into the cell medium for 24 h prior to engraftment in zebrafish embryos to antagonize CXCR4 receptor activation (A). The percentage of live cells after treatment was not significantly different than in the control condition (three independent experiments) (B). The metabolic activity, readout of cell growth, was not significantly affected when increasing concentrations of IT1t were used (C). Pre-treatment *in vitro* caused a reduction in tumor burden at the secondary site *in vivo*, both at 2 dpi (D) and 4 dpi (E). Cancer cell burden increased over time from 2 to 4 dpi in the control group, whereas it remained at comparable levels upon treatment (F). Data set in F is obtained by using the same data points as shown in D and E. Number of larvae is *n*=64 (DMSO) and *n*=75 (IT1t) at 2 dpi and *n*=59 (DMSO) and *n*=56 (IT1t) at 4 dpi. (G) Effect of CXCR4 inhibition on tumor burden over time is shown. Scale bars: 50 µm. Data are mean±s.e.m. from two independent experiments. Statistical analysis: two-tailed, unpaired *t*-test and ANOVA with Bonferroni post-hoc test for datasets with two or more groups respectively. ****P<0.0001; **P=0.005; ns, *P*>0.05.

**Fig. 7.**
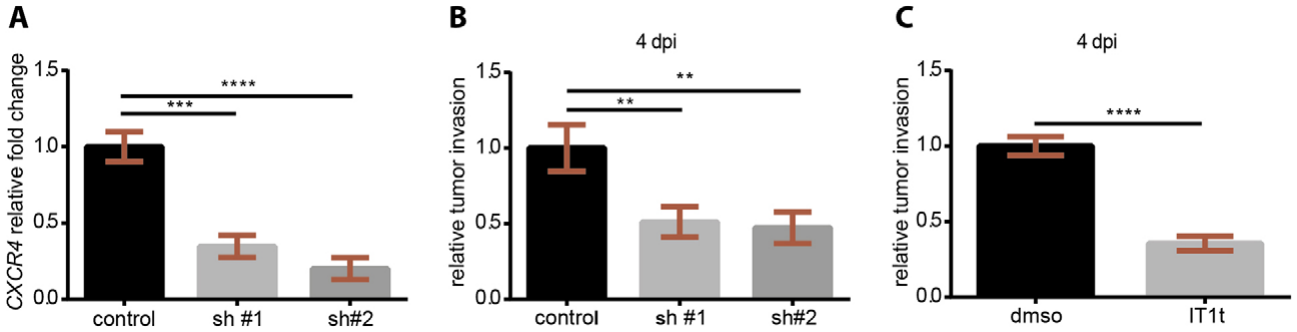
***CXCR4* genetic impairment via RNA interference recapitulates chemical treatment effects on early metastatic events.** (A) *CXCR4* stable knockdown efficiency obtained via shRNA was 66% and 80% for the shRNA #1 (sh#1) and sh#2, respectively. A reduced tumor cell invasion was observed in cell lines carrying one of the *CXCR4* targeting shRNAs (B) as well as upon pre-treatment before engraftment with the CXCR4 antagonist (C). (A,B) One-way ANOVA with Bonferroni post-hoc test: ****P<0.0001, ***P=0.0002, **P<0.01. (C) Un-paired *t*-test: ****P<0.0001.

The use of CXCR4 antagonists as a therapeutic targeted approach to inhibit tumor spreading and the formation of metastases has been introduced in clinical trials for different cancer types. Despite the fact that CXCR4 is highly involved in the establishment of secondary neoplasias, there are no approved FDA drugs to block CXCR4 in TNBC. In the zebrafish xenograft model in which CXCR4-Cxcl12 inter-species communication supports TNBC early metastasis onset, we test whether the isothiourea derivative IT1t, a recently described CXCR4 antagonist (Thoma et al., 2008), displays anti-neoplastic functions. This small molecule is an orthosteric competitor of the CXCL12 N-terminal signaling peptide and it impairs signaling activation by interfering with the docking of the ligand domain to the receptor (Wu et al., 2010). MDA-MB-231-B cells were treated *in vitro* for 24 h and subsequently engrafted in zebrafish embryos. Cells proliferated in treated (20 μM) and untreated conditions (Fig. 6A). Cell survival was not significantly changed: the percentage of live cells was found to be comparable in both groups (97% DMSO, 92% IT1t) (Fig. 6B). To monitor cell viability, a WST-1 (tetrazolium salt) proliferation assay was performed. After a 24 h incubation period with increasing concentrations of IT1t (5, 10 and 20 µM), cancer cell metabolic activity was not changed when compared to vehicle control (Fig. 6C). After pre-treatment (20 µM), engraftment of cells in the blood circulation of 2-dpf zebrafish embryos was performed and tumor burden assessed at the metastatic site at 2 and 4 dpi (Fig. 6D-G). CXCR4 chemical inhibition affected tumor burden, with a 39.5% and 60% reduction at 2 and 4 dpi, respectively (Fig. 6D,E). An increase in tumor burden was found from 2 to 4 dpi for MDA-231-B pre-treated with DMSO, whereas no difference was detected in the IT1t group (Fig. 6F). At 2 dpi, TNBC cells associated to form a secondary mass inside the CV and invaded the tail fin, forming micrometastases at 4 dpi (Fig. 6G, top panel). Blocking CXCR4 *in vitro* impaired tumor mass formation *in vivo*: few cells remained in the CV at 2 dpi and consequently minor invasive events occurred at 4 dpi (Fig. 6G, bottom panel). To phenocopy the suppressive effect of CXCR4 pharmacological inhibition on tumor aggressiveness, we used RNA interference. Stable *CXCR4* knockdown was achieved via lentiviral transduction of two independent *CXCR4* short hairpin RNAs (shRNAs). We confirmed *CXCR4* silencing on a gene expression level, via quantitative PCR (qPCR). Notably, *CXCR4* mRNA levels were decreased in sh#1 and sh#2 compared to scrambled control shRNA, showing a knockdown efficiency of 66% and 80%, respectively (Fig. 7A). Subsequently, xenograft experiments were performed. Tumor cell invasion at the metastatic site was effectively reduced upon *CXCR4* silencing (Fig. 7B), similar to the antagonist IT1t (Fig. 7C). Therefore, using chemical and genetic approaches, we demonstrate that CXCR4 signaling inhibition reduces the formation of TNBC early metastases *in vivo* and describe IT1t as a potential therapeutic for metastatic TNBC.

## DISCUSSION

Metastatic TNBC is a major challenge for biopharmaceutical and clinical research because tumor relapse and cell spreading represent the main cause of death for patients. The development of targeted therapies, in combination with conventional chemotherapy, is an important approach to prolong patient lifetime. Although steps forward in elucidating cancer dissemination have been made, generally the pathogenesis of metastases is not fully understood. Monitoring single tumor cells while crossing the blood vessel boundaries *in vivo* is an optimal scenario to unravel early metastatic events. For this purpose, zebrafish is an advantageous model. The transparency of the embryos and the use of reporter lines make the zebrafish an excellent host to study human tumor cell growth and invasion at early stages (Movie 4). In the last decade the zebrafish xenotransplantation model has been used to study human tumor progression (Konantz et al., 2012) and to discover potential treatments (van der Ent et al., 2014a,b; Veinotte et al., 2014; Zoni et al., 2015). However, the translational validity of a zebrafish xenotransplantation approach has been questioned. Concerns have emerged on possible inter-species crosstalk and lack of species-specific environmental cues. Here, we show that the cross communication between human tumor cells and zebrafish ligands is maintained, because zebrafish Cxcl12 activates human CXCR4 signaling *in vitro* and supports the formation of TNBC early metastases *in vivo*. We found that TNBC cells with high *CXCR4* expression levels exhibit aggressive features in zebrafish, in agreement with findings in patients and other models. In addition to *CXCR4* and *CXCR7* mRNA levels, other differences in gene expression between MDA-MB-231-B and MDA-MB-231 might be present and influence tumor cell behavior. Notably, we proved that CXCR4-linked tumor burden occurred in a zebrafish Cxcl12-dependent manner. The involvement of the human CXCL12 ligand is questioned owing to very low expression levels, in line with the evidence that *CXCL12* expression is higher in non-metastatic breast tumors, compared to metastatic ones (Zhao et al., 2014). However, CXCR4 activation by autocrine mechanisms cannot be fully excluded and *CXCL12* knockdown is required to completely rule out this possibility. Moreover, Cxcr4-expressing macrophages migrate towards human CXCL12, demonstrating that the intercommunication takes place in both directions and confirming the validity of the zebrafish embryo model to study human tumors.

Using the zebrafish xenograft model, we observed that TNBC cells make contact with the endothelium, after inoculation in the blood circulation. Then, tumor-endothelium interaction favors tumor mass formation and, consequently, tumor extravasation and invasion. Interestingly, the invasive process frequently recurs when tumor mass formation and growth are observed at earlier stages, whereas a minimized or absent invasion takes place if no tumor mass is present. This scenario is in accordance with the hypothesis, proposed by Ewing in 1929 (Ewing, 1929), that there is a link between metastasis and organization of the vascular system, stressing the mechanical nature of cancer homing to different sites in the body. At the same time, we could not observe tumor aggregates and invasion phenomena in other tissues along the trunk that are also perfused by the DA and the CV in the zebrafish larvae. The aggressive tumor phenotype occurred mainly in the CHT, a site of hematopoiesis in zebrafish larvae. This is in line with the frequent presence of TNBC metastases in the bone marrow of adult mammals (Shi et al., 2014) and correlates with Cxcl12 ligand expression in zebrafish. In the zebrafish tail region, *cxcl12a* is generally expressed in the CHT (Walters et al., 2010), and expression of *cxcl12a* and *cxcl12b* is normally found in the endothelium of the CV and DA, respectively (Cha et al., 2012). Perhaps both Cxcl12a and Cxcl12b work in concert in sustaining tumor adhesion to the endothelium and consequent tumor burden. Using the zebrafish model, we propose that receptor activation via ligand stimulation and not necessarily in response to a Cxcl12 gradient enhance tumor burden and subsequent invasion. This observation is in line with the ‘seed and soil’ theory proposed by Paget in 1889 (Paget, 1989): tumor cells, the ‘seed’, form a secondary mass in a growth-supportive microenvironment or ‘fertile soil’. Moreover, the preferential growth and invasion in the CHT partially explain why the bone clone and not the parental line MDA-MB-231, more commonly used in other animal models, showed aggressive features in the zebrafish embryo. In conclusion, our data are in agreement with previous theories (Fidler, 2003) that both mechanical and microenvironment-related factors contribute to tumor mass formation and cell invasion to ultimately initiate the metastatic process.

Attempts in the clinic have been made to pharmaceutically interfere with CXCR4-CXCL12 signaling. AMD3100 (plerixafor), the most commonly used drug to inhibit the receptor CXCR4, is currently in clinical trials for glioma, leukemia, Ewing sarcoma, neuroblastoma and brain tumors and is already FDA-approved for Non-Hodgkin's lymphomas and multiple myeloma. However, long-term secondary effects and mobilization of hematopoietic stem cells have been registered. Alternatively, CXCL12 targeting agents are currently in clinical trials and under investigation (Scala, 2015). IT1t is an orally available isothiourea compound that antagonizes CXCR4 activation with high specificity and potency, as shown in the Ca^2+^ flux assay, inhibition of X4-HIV attachment and whole-blood actin polymerization assay in rats (Thoma et al., 2008). Moreover, residues involved in IT1t binding to CXCR4 are not conserved in CXCR7 (Yoshikawa et al., 2013). We showed for the first time that CXCR4 inhibition via IT1t results in a reduction of early metastasis formation *in vivo*. A reduction in tumor invasion as well as tumor mass formation was observed at 4 dpi in zebrafish larvae, after 24 h pre-incubation *in vitro*. We propose that the treatment affects ligand sensing *in vivo*, therefore affecting the ability of tumor cells to survive in the blood circulation, colonize the CHT, and to make contact with the endothelium to subsequently proliferate and extravasate, initiating early metastatic events. Cancer cell proliferation can occur inside blood vessels (Hanahan and Weinberg, 2011) and extravasation events are linked to tumor-cell–endothelium interaction (Stoletov et al., 2010). Moreover, highly adherent tumor cells have been reported to have stem-cell-like features (Bansal et al., 2014). Hence, it is not to be excluded that TNBC cells that initiate early metastatic events in our model have stem-like properties and might express high levels of CXCR4.

In conclusion, the zebrafish xenotransplantation model, in which inter-species crosstalk is maintained, has provided new insight into the metastatic events associated with TNBC and into the employment of a potential compound to limit CXCR4-dependent tumor early metastases.

## MATERIALS AND METHODS

### Zebrafish husbandry

Zebrafish lines were maintained according to standard protocols, described in zfin.org, and handled in accordance with the Dutch animal welfare regulations and the EU Animal Protection Directive 2010/63/EU.

### Zebrafish lines

In the present study, the reporter lines *Tg(kdrl:EGFP)^s843^* (Jin et al., 2005), *Tg(mpeg1:mCherry)^UMSF001^* (Bernut et al., 2014) and *Tg(mpeg1:EGFP)^gl22^* (Ellett et al., 2011) were used to monitor tumor cell behavior, macrophage recruitment and motility, respectively. Mutant lines used were *cxcl12a^t30516^* (*medusa*) (Valentin et al., 2007), *cxc12b^mu100^* (Bussmann et al., 2011) and *cxcr4b^t26035^* [*odysseus* (*ody*)] (Knaut et al., 2003). *cxcl12a^t30516/t30516^* (*cxcl12a^−/−^*) and *cxcr4b^t26035/t26035^* (*cxcr4b^−/−^*) mutants were identified, before raising, for incomplete migration of the lateral line primordium at the larval stage. Adult fins were clipped and DNA extraction for genotyping was performed. Genotype identification was carried out using a KASP assay. The following primers were used: A1 (reverse) 5′-CTGTGTTGACTGTGGAACGGCAC-3′, A2 (reverse) 5′-CTGTGTTGACTGTGGAACGGCAT-3′ and C1 (forward) 5′-AGCCAAGCCCATCAGCCTGGTA-3′ for *cxcl12a*; A1 (forward) 5′-GTGCTGGTGTCGCTCCACC-3′, A2 (forward) 5′-GTGCTGGTGTCGCTCCACG-3′ and C1 (reverse) 5′-AACTTGATCTCTCGGATGCTCCGTT-3′ for *cxcl12b*; and A1 (reverse) 5′-TGACGGTGGTCTTCAGTGCCTT-3′, A2 (reverse) 5′-TGACGGTGGTCTTCAGTGCCTA-3′ and C1 (forward) 5′-CAAGAACTCCAAGGGTCAGACTCTA-3′ for *cxcr4b*. KASP assay results were confirmed by sequencing, using the following primers: 5′-AGGATGCTGTTCCGTTTTAC-3′ (forward) and 5′-TGTGTGTGTCTGACTAAGCA-3′ (reverse) for *cxcl12a*; and 5′-AAGCCCATCAGTCTGGTGGAGAGG-3′ (forward) and 5′-GTGCCCTTTGTCTGGTGTAACCTG-3′ (reverse) for *cxcl12b*. Primers for *cxcl12b^−/−^* (Bussmann et al., 2011) and *cxcr4b^−/−^* (Miyasaka et al., 2007) identification were previously described and used for sequencing. For experiments, *cxcl12a^−/−^/cxc12b^+/−^/Tg(kdrl:EGFP)^s843^* were in-crossed and double mutants were identified based on impaired connection of the hypobranchial artery branches to the first aortic arch at 6 dpf. *Cxcl12a^−/−^* siblings, wild-type or heterozygote for *cxcl12b^mu100^*, were considered as control groups.

### Cell culture

The breast cancer cell line MDA-MB-231 [American Type Culture Collection (ATCC)], the derived bone clone MDA-MB-231-B and MCF-7 were cultured in DMEM complemented with 10% fetal calf serum (FCS) and grown at 37°C and 5% CO_2_. pLenti-tdtomato plasmid was introduced via lentiviral transduction in MDA-MB-231, whereas MDA-MB-231-B cells stably expressed dsRed fluorescent protein. Blasticidin and G418 were used to select cell clones that were tdtomato- or dsRed-positive, respectively. Cell lines were regularly tested for mycoplasma with the Universal Mycoplasma Detection Kit (30-1012k, ATCC).

### Proliferation assay

MDA-231-B tumor cells were treated with increasing concentrations (5, 10 and 20 μM) of CXCR4 antagonist IT1t (239821, Calbiochem) to assess cell growth. A total of 30,000 cells were seeded in a single well of a 96-well plate. The following day, inhibitor treatment was carried on for 24 h. WST-1 tetrazolium salt (05015944001, Roche) was added into the cell medium and absorbance of the reduced by-product was measured to quantify cell viability.

### RNA interference

*CXCR4* stable knockdown in MDA-MB-231-B was obtained using shRNA containing constructs derived from Sigma MISSION library [TRCN0000004054 (or #1): 5′-CTTTGTCATCACGCTTCCC-3′ and TRCN0000004056 (or #2): 5′-GAATCACGTAAAGCTAGAA-3′]. Lentivirus virions were produced by transfecting HEK293T cells with pKLO1-puro plasmid (containing the *CXCR4*-targeting shRNA or a non-mammalian shRNA control), pCMV-VSV-G (envelope plasmid), pMDLg-RRE (gag and pol elements) and pRSV-REV (rev or HIV1gp6) as packaging vectors. Plasmid mix was added to cell medium together with CaCl_2_ and incubated for 18-20 h. Virus-containing supernatant was collected 48 and 72 h post-transfection and virus concentration was measured using Lenti-X™ p24 Rapid Titer Kit (Clontech). For transduction, MDA-MB-231-B cells were seeded in a 24-well plate (25,000 cells/well) and lentiviruses [1-3 multiplicity of infection (MOI)] added together with polybrene overnight (O.N.). Cells were cultured with complete medium containing 1 µg/ml puromycin for four passages and samples were collected for RNA isolation.

### RNA isolation, cDNA synthesis and qPCR

RNA was isolated using a High Pure RNA Isolation Kit (Roche). After DNAase treatment, complementary DNA (cDNA) synthesis was performed (i-Script™ cDNA Synthesis Kit, Bio-Rad) and expression levels were measured by qPCR (iQ™ SYBR^®^ Green Supermix, Bio-Rad). Relative fold changes of gene expression were calculated using the ΔΔCt method. *CXCR4* primers (Fw: 5′-CAGCAGGTAGCAAAGTGACG-3′; Rv: 5′-GTAGATGGTGGGCAGGAAGA-3′; amplicon size: 150 bp) were kindly provided by Dr S. B. Geutskens (LUMC, Leiden, The Netherlands) and *CXCL12* (Fw: 5′-CACATCTAACCTCATCTTC-3′; Rv: 5′-GACTTACTCTTCACATAGC-3′; amplicon size: 180 bp) primers were described in Costantini et al. (2013). *GAPDH* was used as housekeeping gene (Fw: 5′-AATCCCATCACCATGTTCCA-3′; Rv: 5′-TGGACTCCACGACGTACTCA-3′; amplicon size: 160 bp) (van der Vaart et al., 2014).

### Ca^2+^ flux assay

MDA-MB-231-B and MDA-MB-231 cells (5×10^4^ to 1×10^5^) were seeded in uncoated µ-Dish^35mm^ ibidi dishes (81156, ibidi) to adhere overnight. Adherent cells forming a 50% confluent monolayer were pre-incubated for 30 min at 37°C with 1-10 µM cell permeant calcium sensor Fluo-4, AM (F14217, Invitrogen). Cells were kept in Dulbecco's phosphate buffered saline (DPBS) until and during imaging. Ca^2+^ flux was measured upon stimulation with recombinant human CXCL12-α (300-28A, Peprotech) (100-300 nM), zebrafish CXCL12a (500 nM) or CXCL12b (100 nM) ligands (Boldajipour et al., 2011) by fluorescent signal imaging, using an Axiovert200 microscope (Zeiss, Germany) combined with a spinning disk unit (CSU-X1, Yokogawa, Japan) and a CCD camera (iXon 897, Andor, UK). Time-lapse imaging was performed using a 20× objective with a laser illumination at 488 nm (Crystal), at a 200 ms or 1 s time interval. Image analysis was performed using self-written algorithms in MatLab (MathWorks Inc., USA).

### Inoculum preparation for engraftment and xenotransplantation

Cell suspension was prepared once cells had grown to a 70-80% confluent monolayer. After detachment with trypsin-EDTA (30-2101, ATCC^®^), tumor cells were washed once in complete medium and twice in DPBS (GIBCO^®^ by Life Technologies). Centrifugation steps were performed for 5 min at 200 ***g*** (Eppendorf 5702). 2% PVP40 (polyvinylpyrrolidone-40) was used for the final cell suspension. 2-day-old zebrafish embryos, manually dechorionated and treated with 0.003% PTU (1-phenyl-2-thiourea, Sigma-Aldrich) at 24 hpf, were anesthetized using 0.02% Tricaine (MS-222) and transferred in a Petri dish with a 1.5% agarose coating layer. Cell suspension was loaded in a glass capillary, prepared using a Flaming/Brown micropipette puller (model P-97, Sutter Instrument Co.). Forceps were used to cut the end of the needle and injection tests were performed in a drop of water to set pressure and time parameters, in order to engraft 300-500 cells. Tumor cells were inoculated in the blood circulation, via the duct of Cuvier. Engrafted embryos were transferred to a new Petri dish and kept at 34°C. Embryos were checked 3- to 5-h post implantation (hpi) for correct engraftment and the ones showing tumor cells in the blood circulation were selected for experiments.

### Microscopy and phenotype assessment *in vivo*

Tumor burden of early metastases, extravasation and tissue invasion were assessed via imaging of the tail fin region, in proximity of the CHT, at 2 and 4 dpi. In order to quantify tumor burden, single-embryo pictures were acquired. Cell aggregates inside the blood vessels, as well as extravasating and invading single cells, were included in the analysis of tumor burden and comprehended in the definition of early metastases and micrometastases. Tumor cell extravasation and invasion were also quantified separately from intravascular tumor mass by counting the number of cells per embryo and acquiring representative micrographs. A Leica MZ16FA fluorescent microscope coupled to a DFC420C camera was used. GFP and dsRed channels were overlaid in LAS AF Lite software and snapshots were analyzed in Image-Pro Analyzer 7.0 (Media Cybernetics). For each larva, tumor burden was calculated based on the number of objects multiplied by mean area and mean intensity, generated with a macro designed by H. de Bont (Toxicology, LACDR, Leiden University) and previously used to quantify tumor migration and proliferation (Ghotra et al., 2012; van der Ent et al., 2014b). In each micrograph, larvae are shown in lateral view and oriented head (left)-to-tail (right), as shown by representative cartoons.

### Chemokine injection, L-P staining and *in silico* analysis

Human CXCL12-α (0.4 mg/ml) (300-28A, Peprotech) or zebrafish Cxcl11aa (1.5 mg/ml) (Torraca et al., 2015) chemokines, or water control (1 nl), were injected in the hindbrain ventricle (HBV) of 30-32 hpf embryos. Sample fixation was done at 3-3.5 hpi with 4% paraformaldehyde (PFA) (O.N. at 4°C or for 3 h at room temperature). Macrophages were counted as mpeg^+^ cells in the *Tg(mpeg1:mCherry)^UMSF001^* line and L-plastin (L-P)^+^/Tyramide Signal Amplification (TSA)^−^ cells when immunohistochemistry was performed, as described previously (Cui et al., 2011; Loynes et al., 2010). Images were acquired using a Leica TCS SPE confocal microscope with an HC PL FLUOTAR 10× DRY objective (0.30 N.A.). In each micrograph, embryos are shown in lateral view and oriented head (left)-to-tail (right) and injection site shown by schematic drawing. Human and zebrafish CXCL12/Cxcl12 and CXCR4/Cxcr4 sequences were obtained in UniProt (The UniProt Consortium, 2015) and aligned in ClustalW (Goujon et al., 2010; Larkin et al., 2007). Specific domains of the human proteins for ligand binding and receptor activation were reported in UniProt.

### Statistical analysis

Unpaired, two-tailed *t*-test was used to compare the means of two groups and Welch's correction applied when variances were significantly different (*P*<0.05). For datasets of three or more groups, one-way ANOVA with Bonferroni post-hoc test was performed. Raw or normalized data are mean±s.e.m. of pooled data points from at least two independent experiments. Statistics were performed with GraphPad Prism 6.
